# The role of adatoms in chloride-activated colloidal silver nanoparticles for surface-enhanced Raman scattering enhancement

**DOI:** 10.3762/bjnano.9.208

**Published:** 2018-08-22

**Authors:** Nicolae Leopold, Andrei Stefancu, Krisztian Herman, István Sz Tódor, Stefania D Iancu, Vlad Moisoiu, Loredana F Leopold

**Affiliations:** 1 Faculty of Physics, Babeş-Bolyai University, Kogalniceanu 1, 400084 Cluj-Napoca, Romania; 2 Faculty of Food Science and Technology, University of Agricultural Sciences and Veterinary Medicine, Manastur 3-5, 400372 Cluj-Napoca, Romania

**Keywords:** chloride activation, electronic coupling, photoreduction, silver nanoparticles, SERS-active sites, SERS switch-on effect

## Abstract

Chloride-capped silver nanoparticles (Cl-AgNPs) allow for high-intensity surface-enhanced Raman scattering (SERS) spectra of cationic molecules to be obtained (even at nanomolar concentration) and may also play a key role in understanding some fundamental principles behind SERS. In this study, we describe a fast (<10 min) and simple protocol for obtaining highly SERS-active colloidal silver nanoparticles (AgNPs) with a mean diameter of 36 nm by photoconversion from AgCl precursor microparticles in the absence of any organic reducing or capping agent. The resulting AgNPs are already SERS-activated by the Cl^−^ ions chemisorbed onto the metal surface where the chloride concentration in the colloidal solution is 10^−2^ M. Consequently, the enhanced SERS spectra of cationic dyes (e.g., crystal violet or 9-aminoacridine) demonstrate the advantages of Cl-AgNPs compared to the as-synthesized AgNPs obtained by standard Ag^+^ reduction with hydroxylamine (hya-AgNPS) or citrate (cit-AgNPs). The results of SERS experiments on anionic and cationic test molecules comparing Cl-AgNPs, hya-AgNPs and cit-AgNPs colloids activated with different amounts of Cl^−^ and/or cations such as Ag^+^, Mg^2+^ or Ca^2+^ can be explained within the understanding of the adatom model – the chemisorption of cationic analytes onto the metal surface is mediated by the Cl^−^ ions, whereas ions like Ag^+^, Mg^2+^ or Ca^2+^ mediate the electronic coupling of anionic species to the silver metal surface. Moreover, the SERS effect is switched on only after the electronic coupling of the adsorbate to the silver surface at SERS-active sites. The experiments presented in this study highlight the SERS-activating role played by ions such as Cl^−^, Ag^+^, Mg^2+^ or Ca^2+^, which is a process that seems to prevail over the Raman enhancement due to nanoparticle aggregation.

## Introduction

The most common surface-enhanced Raman scattering (SERS) substrate is the silver colloid obtained by Ag^+^ reduction with citrate (cit-AgNPs), proposed by Lee and Meisel in 1982 [1]. Although the preparation of cit-AgNPs requires boiling conditions, this colloid is often preferred due to its high preparation success rate. Another often used SERS substrate is the silver colloid obtained by Ag^+^ reduction with sodium borohydride [2], or, more recently, with hydroxylamine hydrochloride (hya-AgNPs) [3]. As we will show in the present study, the higher Raman enhancement of the hya-AgNPs compared to as-synthesized cit-AgNPs arises from the presence of chemisorbed Cl^−^ ions, which form SERS-active sites on the silver nanoparticle (AgNP) surface. More precisely, the as-synthesized hya-AgNP colloidal solution already contains Cl^−^ ions in a concentration of 2.4 mM due to the addition of the hydrochloride form of hydroxylamine to the reaction mixture, from which a small part of Cl^−^ ions become chemisorbed onto the AgNP surface, thus generating the SERS-active sites.

Silver halides are a class of light-sensitive materials that have been extensively used for photographic films. Gao et al. [4] were the first to report SERS spectra acquired from a dye adsorbed onto silver nanoparticles formed at the surface of silver chloride particles suspended in water. The photoconversion of silver salts to metallic silver particles is the basic principle of film photography: silver ions in silver halide particles are photoreduced by the transfer of electrons from halide ions [4–5].

Chloride anions are often added to metal colloids to enhance the Raman signal of the analytes. This phenomenon can be explained by two distinct mechanisms: the electromagnetic mechanism and the chemical effect. Each mechanism is believed to contribute complementary to the Raman scattering enhancement of the adsorbate in SERS spectroscopy. When the SERS effect is explained in terms of the electromagnetic mechanism, an aggregating effect of the colloidal nanoparticles is attributed to the chloride ions [6], whereas in the chemical mechanism, a so-called activation effect is attributed to the chloride ions [7].

The electromagnetic mechanism arises due to enhanced local optical fields at the site of the molecule situated in the close proximity of the metal surface. This local enhancement is determined by the resonant excitation of surface plasmons, which are collective oscillations of the conducting electrons in the metallic nanoparticles (also called surface plasmon resonances) [8]. Although this model does not require a chemical contact, the magnitude of the electromagnetic field outside the particle decreases with the third power of radial distance [9]. This means that the field enhancement of Raman scattering decreases strongly with the distance from the metal surface.

Chloride ions, when used at concentrations higher than 0.1 M, induce the aggregation of the metal colloids. The addition of Cl^−^ ions to the colloidal solution reduces the absolute zeta potential value of the nanoparticles, leading to a decrease in the stability of the colloid and therefore the aggregation of nanoparticles. Consequently, according to literature, electromagnetic hot spots, i.e., sites of highly increased field strength, are generated in the gaps between adjacent nanoparticles [10–11]. The maximal values for electromagnetic enhancement are on the order of 10^6^–10^7^ for isolated metal nanoparticles [8].

The chemical mechanism of Raman signal enhancement is based on a metal–molecule electronic transition [8,12]. The electronic coupling of the molecule with the metal surface of the nanoparticle leads to a significant increase in the Raman cross-section of the adsorbed molecule. The charge-transfer electronic transition can be explained as follows [8,12–14]:


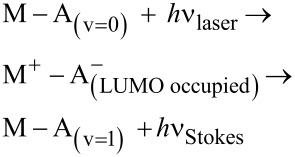


The chemical mechanism of SERS involves the absorption of a photon and the excitation of an electron from the Fermi level of the metallic nanoparticle (M) to the LUMO orbital of the adsorbed molecule (A). Thus, an excited state is achieved (M^+^ − A^−^). If the molecule remains vibrationally excited during the transition of the electron from the excited state of the adsorbed molecule back to the metallic nanoparticle, then the transition is accompanied by the emission of a Raman photon (*h*ν_Stokes_).

As proposed by Otto and co-workers [12–13], adsorbed atoms (adatoms) facilitate the metal–molecule electronic transfer, providing pathways for these transitions. Thus, according to the adatom model of SERS, the Raman enhancement effect appears only if the adsorbed molecule is bound to an adatom [13,15–16]. The adsorption of chloride ions will lead to the formation of additional adatoms on the surface of the nanoparticles, thus activating the metal surface of the nanoparticle [12–13,17].

Likewise, the formation of complexes of the type Ag^+^–phthalazine–halide anion on AgNP surfaces has also been proposed by Muniz-Miranda and Sbrana [18].

In contrast to the electromagnetic mechanism, the chemical effect requires an electronic contact between adsorbate and metal surface [14]. The enhancement factor for the chemical effect is estimated to be between 10–100 [8].

In this study, we describe the synthesis of a stable colloidal solution of AgNPs capped only with Cl^−^ ions, where the chemisorbed part of the Cl^−^ ions forms the SERS-active sites for cationic molecules. Furthermore, the addition of cations such as Ag^+^, Mg^2+^ or Ca^2+^ increases the number of SERS-active sites on the AgNP surface by mediating the chemisorption of Cl^−^ ions. Hence, cationic dye molecules are electronically coupled to the silver surface at these SERS-active sites, leading to high-intensity SERS spectra. The appearance of the SERS effect only after the electronic coupling of the analyte to the Ag surface explains why the citrate surfactant is not SERS active in the as-synthesized cit-AgNPs. Nevertheless, the SERS spectrum of the citrate surfactant can be turned on by the chemisorption of the citrate molecules onto the metal surface, mediated by cations such as Ag^+^, Mg^2+^ or Ca^2+^. In general, all the experiments performed in this study prove that the SERS effect is turned on only after the electronic coupling of the analyte to the Ag surface at SERS-active sites.

## Results and Discussion

### Cl-AgNP synthesis monitoring

**Figure 1:**
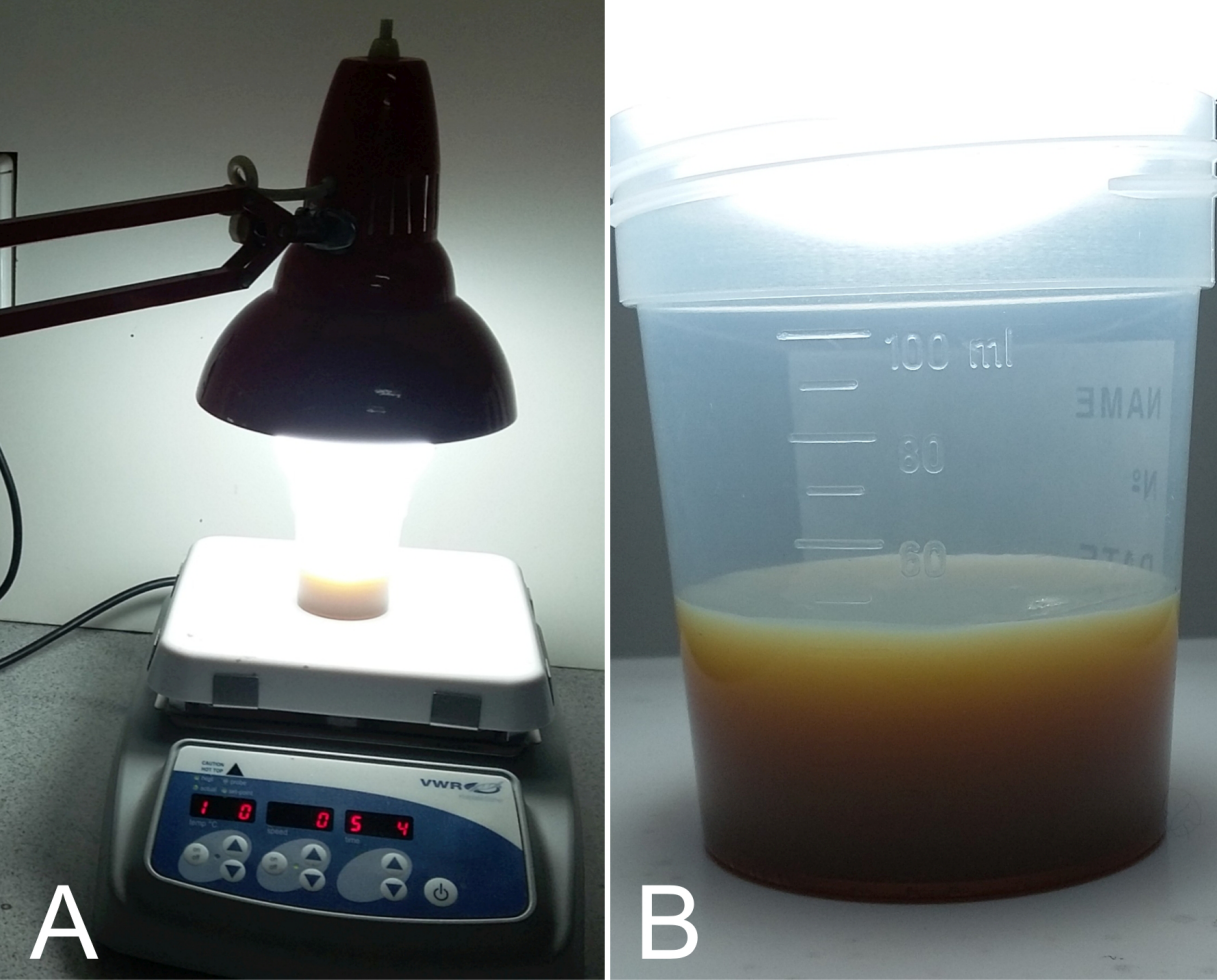
(A) Picture showing the experimental arrangement for the synthesis of Cl-AgNPs. A desk lamp with an LED bulb was placed above the beaker containing the reaction mixture. (B) Picture of the colloidal solution of Cl-AgNPs obtained after 10 min of light exposure.

Cl-AgNPs can be synthesized within 10 min of light exposure. Figure 1 shows a picture of the experimental setup used for the synthesis of Cl-AgNPs (Figure 1A) and a picture of the resulting colloid (Figure 1B).

After the first 3 min of light exposure, the solution turned pale yellow; after 5 min of light exposure, an intense gray-brown color indicated a high concentration of AgNPs in the solution. Also, at this time, gaseous reaction products were observed, probably due to the O_2_ released during the decomposition of hydrogen peroxide. No further color changes could be visually observed after 5 min of light exposure, but the reaction mixture was further exposed to light for a total of 15 min and afterwards stored in the dark for 24 h.

A slightly faster synthesis was observed when covering the beaker in aluminum foil, due to the local increase in light intensity. Also, we observed that a LED bulb with a “cool light” emission spectrum led to a faster synthesis compared to an equivalent power “warm light” emitting bulb. Nonetheless, similar results were obtained when using a lower power LED bulb (12 W, 6400 K, cool white, 960 lm).

However, in most of the reported studies on silver nanoparticles synthesized by photoreduction, the photosynthesis was performed in the presence of organic stabilizing agents such as citrate [19] or poly(*N*-vinylpyrrolidone) [20–21]. The photoreduction of AgCl crystals has also been previously reported; however, the photoconversion of AgCl to silver nanoparticles was performed in the presence of stabilizing agents, such as proteins [22] or DNA [23–24]. Compared to these studies, the most important novelty of our protocol is represented by the rapid synthesis in addition to the absence of any organic capping agent.

**Figure 2:**
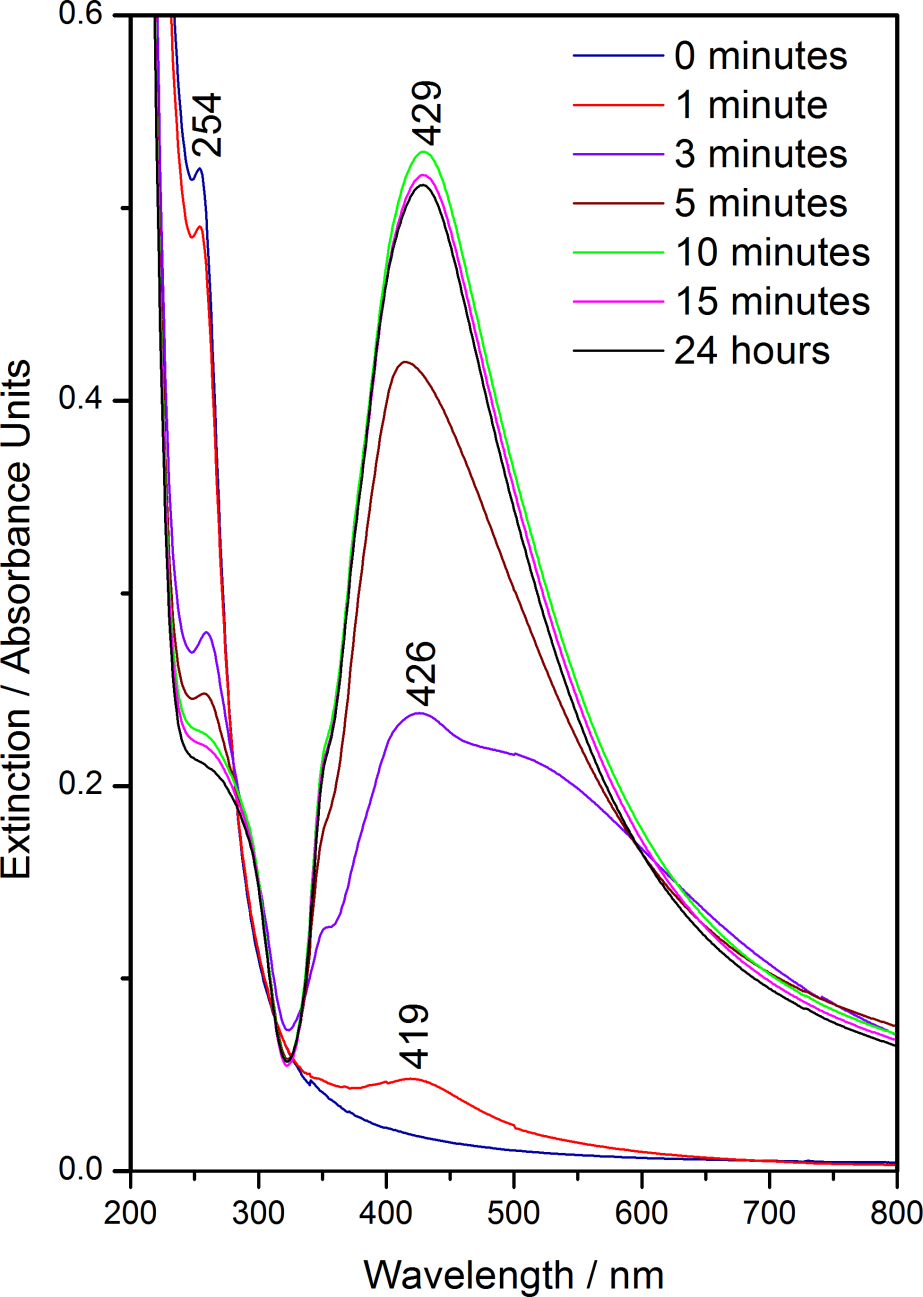
UV–vis extinction spectra obtained during the Cl-AgNP synthesis, recorded after 0, 1, 3, 5, 10 and 15 min of light exposure to the reacting mixture in addition to 24 h after the colloid synthesis.

The UV–vis spectral monitoring of the Cl-AgNPs during the synthesis process (Figure 2) shows the photoconversion of AgCl microparticles to AgNPs in the first 10 min of light exposure.

Mixing the silver nitrate and sodium chloride in the reacting solution resulted in the generation of a flocculent precipitate of AgCl. The presence of AgCl particles in the solution was evidenced in the UV–vis spectra as an intense absorption band at 254 nm (Figure 2) [25–26]. It should be mentioned that none of the reagents measured separately showed an absorption band at this wavelength and that the AgCl absorption band can also be observed in the UV–vis spectrum of a mixture of only AgNO_3_ and NaCl (Supporting Information File 1, Figure S1).

During the exposure of the reaction mixture to light, the intensity of the 254 nm band due to AgCl absorption decreases, while the plasmonic band intensity of the nascent AgNPs increases. The UV–vis spectrum recorded after 1 min shows a very low intensity plasmonic band at 419 nm, indicating the formation of only a small number of metallic silver nanoparticles, as well as a slight decrease in the intensity of the AgCl band.

After 3 min of light exposure, the UV–vis absorption spectrum of the reaction mixture shows a large plasmonic band with a maximum at 426 nm and a wide shoulder around 550 nm, indicating a high polydispersity of the formed silver nanostructures. Simultaneously, a decrease in the intensity of the AgCl absorption band at 254 nm is observed, whereby the intensity of the AgCl band is then comparable to that of the plasmonic band. However, after 5 min of light exposure, the plasmon resonance band became narrower, showing a typical shape for silver colloids.

Between 5 and 10 min of light exposure, the intensity of the plasmonic band at 429 nm reaches its maximum, and no further increase of the plasmonic band intensity could be observed between 10 and 15 min of light exposure. Furthermore, within 24 h from the synthesis of the colloid, no notable changes in the UV–vis spectrum were observed. Consequently, we can conclude that the colloidal solution is synthesized within the first 10 min of light exposure.

Intense light exposure is critical for the synthesis of Cl-AgNPs, since the reaction does not proceed in the absence of an intense light source, regardless of the amount of hydrogen peroxide added in the reaction mixture.

It should be mentioned that the formation of stable, SERS-active Cl-AgNP colloids can also be obtained in the absence of hydrogen peroxide, but in this case, the complete synthesis of the silver colloids takes several hours. The UV–vis absorption monitoring of the Cl-AgNP synthesis obtained in the absence of hydrogen peroxide is presented in Supporting Information File 1, Figure S2A, whereas the SERS activity of the Cl-AgNPs tested after different times of light exposure is shown in Figure S2B (using crystal violet as an analyte).

**Figure 3:**
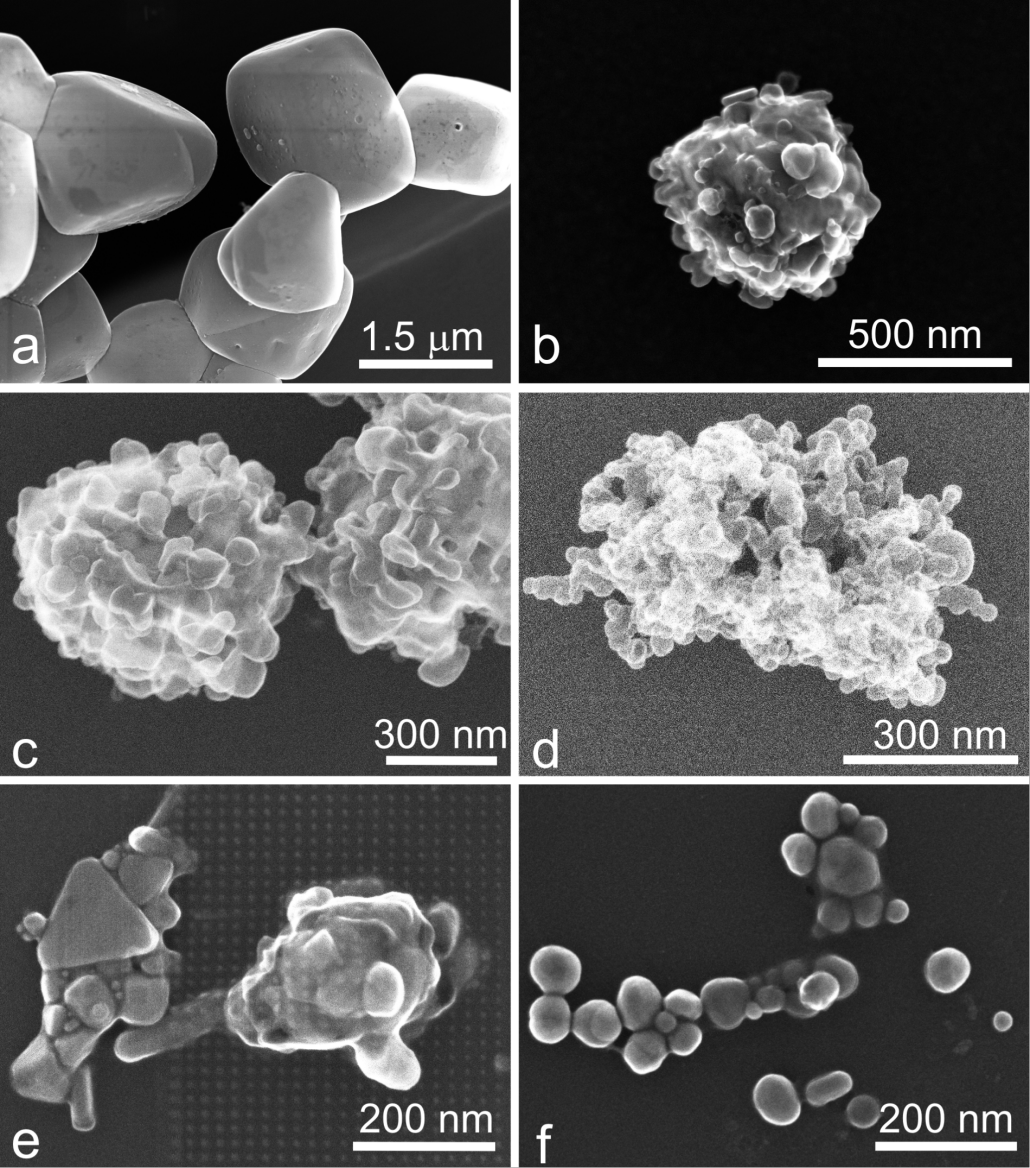
SEM micrographs showing different stages during the photoconversion of AgCl microparticles (a) to AgNPs (f).

The formation of AgNPs from AgCl precursor microparticles can be also followed in the scanning electron microscopy (SEM) micrographs depicted in Figure 3.

Figure 3a shows 1–2 μm AgCl particles formed after mixing silver nitrate and sodium chloride in the reacting solution.

During the photoreduction process, the surface of the AgCl particles becomes covered with clusters of Ag atoms, which was also reported by Wang et al. [27].

The silver atom clusters grow continuously during light exposure, leading to the formation of AgNPs on the surface of AgCl microparticles, as shown in Figure 3b–d. Figure 3e,f shows the AgNPs obtained after the full photoconversion from AgCl precursor microparticles, where the majority of the Cl-AgNPs in Figure 3f have a diameter between 30 and 40 nm. A mean diameter of 36 nm was estimated from the electron microscopy micrographs.

### SERS spectra of cations

**Figure 4:**
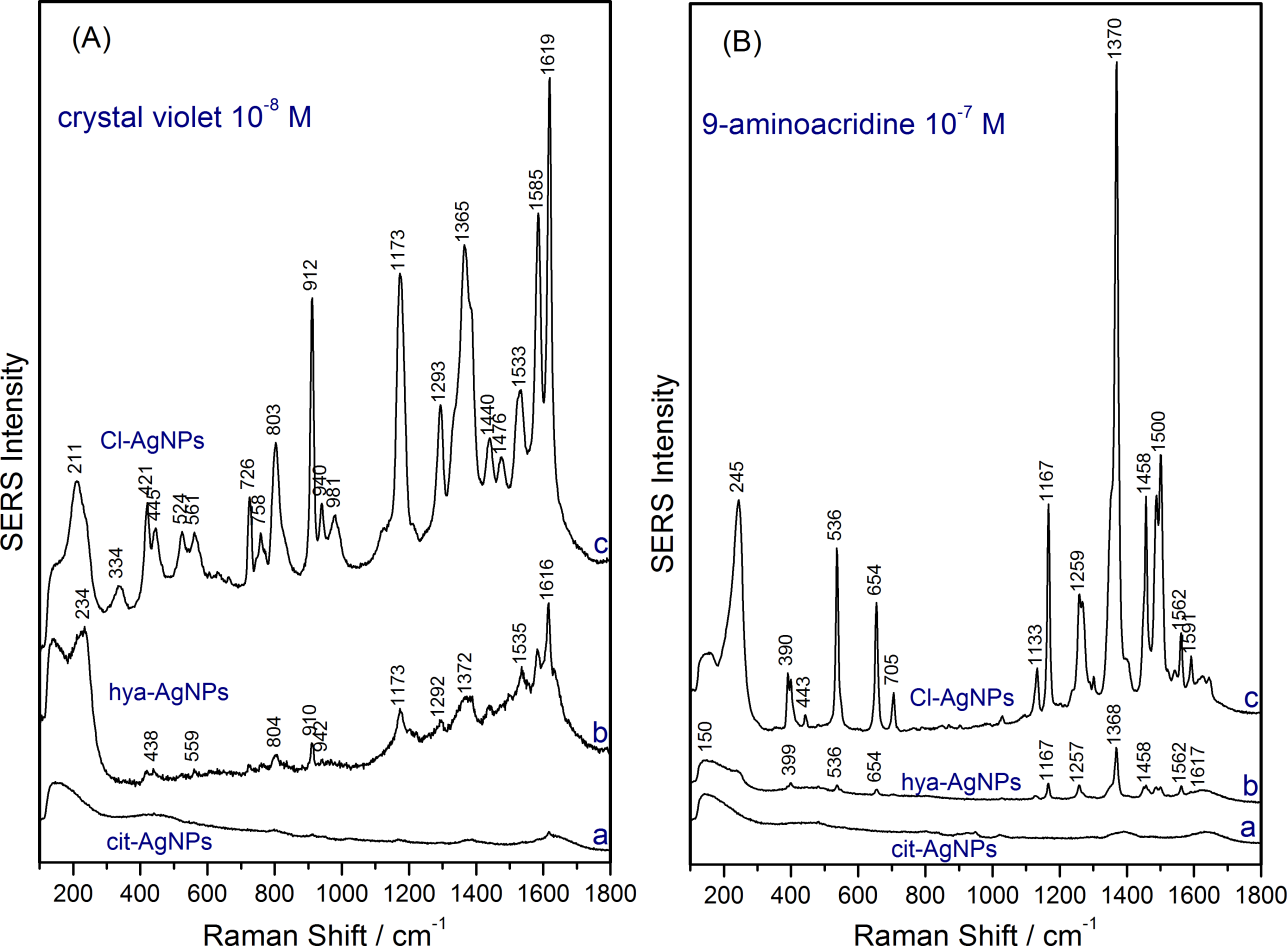
(A) SERS spectra of crystal violet 10^−8^ M and (B) 9-aminoacridine 10^−7^ M obtained using three different silver colloidal solutions as substrate: cit-AgNPs (a), hya-AgNPs (b), and Cl-AgNPs (c), as indicated in the figure.

To highlight the role of the Cl^−^ ions in the generation of SERS-active sites on the silver surface and chemisorption of cationic analyte molecules, we performed comparative SERS experiments using Cl-AgNPs, cit-AgNPs and hya-AgNPs. Crystal violet chloride and 9-aminoacridine hydrochloride monohydrate were used as test analytes. Figure 4 shows comparatively the SERS spectra of the two cationic dyes obtained using the three silver colloids.

By far, the Cl-AgNPs displayed the highest SERS intensity for both cationic dyes, followed by hya-AgNPs, which showed only low intensity SERS bands, and finally cit-AgNPs, for which no SERS bands were visible. The higher SERS enhancement for the Cl-AgNPs was expected, since this colloid contains around 4 times more Cl^−^ ions (10 mM) compared to hya-AgNPs (2.4 mM). However, the SERS enhancement of Cl-AgNPs is more than one order of magnitude higher than that of hya-AgNPs. This finding can be explained within the adatom model, which states that the enhancement is not due to the “free” Cl^−^ ions, but only due to chemisorbed Cl^−^ ions (adatoms), which form SERS-active sites on the AgNPs. Hence, the chemisorbed Cl^−^ ions mediate the electronic coupling of the cationic molecules by forming Cl^−^–adsorbate surface complexes [18]. Consequently, the fact that the highest Raman enhancement was obtained using Cl-AgNPs as substrate can be explained by the greater number of SERS-active sites in the case of this colloid. The SERS activation effect of chloride was reported in several studies. Futamata and Maruyama showed that Cl^−^ ions induce the electronic interaction between silver nanoparticles and rhodamine 6G (R6G), leading to intense SERS spectra [28]. Doering and Nie highlighted the activating effect of halide ions such as Cl^−^, Br^−^ or I^−^ on single-particle SERS [29]. In addition, they observed that the selective removal of Ag^+^ by thiosulfate (a fixative used in black-and-white photography to dissolve residual Ag^+^ cations) leads to a dramatic decrease in the number of SERS-active nanoparticles, underlining thus the need for Ag^+^ cations for the generation of SERS-active sites on the nanoparticle surface [28–29].

When using the cit-AgNPs as a SERS substrate, no SERS spectra of the dye molecules could be obtained since the Lee–Meisel colloid does not contain any chloride ions that could form SERS-active sites. Small amounts of Cl^−^ ions (10^−8^–10^−7^ M) are nonetheless present in the solution, since the test molecules were added as chloride salts, but not enough to observe a SERS effect.

However, after adding 0.1 M NaCl to the cit-AgNPs or hya-AgNPs, intense SERS spectra of crystal violet or 9-aminoacridine were obtained (data not shown). We suppose that besides aggregating the colloid upon Cl^−^ addition, the increase in the SERS intensity of the test analytes is also explained by the generation of more SERS-active sites on the silver surface of the cit-AgNPs or hya-AgNPs.

In the next section we will show that cations such as Ag^+^, Mg^2+^ or Ca^2+^ promote the chemisorption of Cl^−^ ions, thereby generating SERS-active sites for cationic dye molecules. Thus, instead of adding additional Cl^−^ ions, the SERS intensity of the analyte can be increased by adding cations such as Mg^2+^ or Ca^2+^, which promote the chemisorption of more Cl^−^ onto the silver surface from the total amount of “free” Cl^−^ ions present in the hya-AgNPs or Cl-AgNP colloids.

### AgNP activation for obtaining SERS spectra of anions and cations

**Figure 5:**
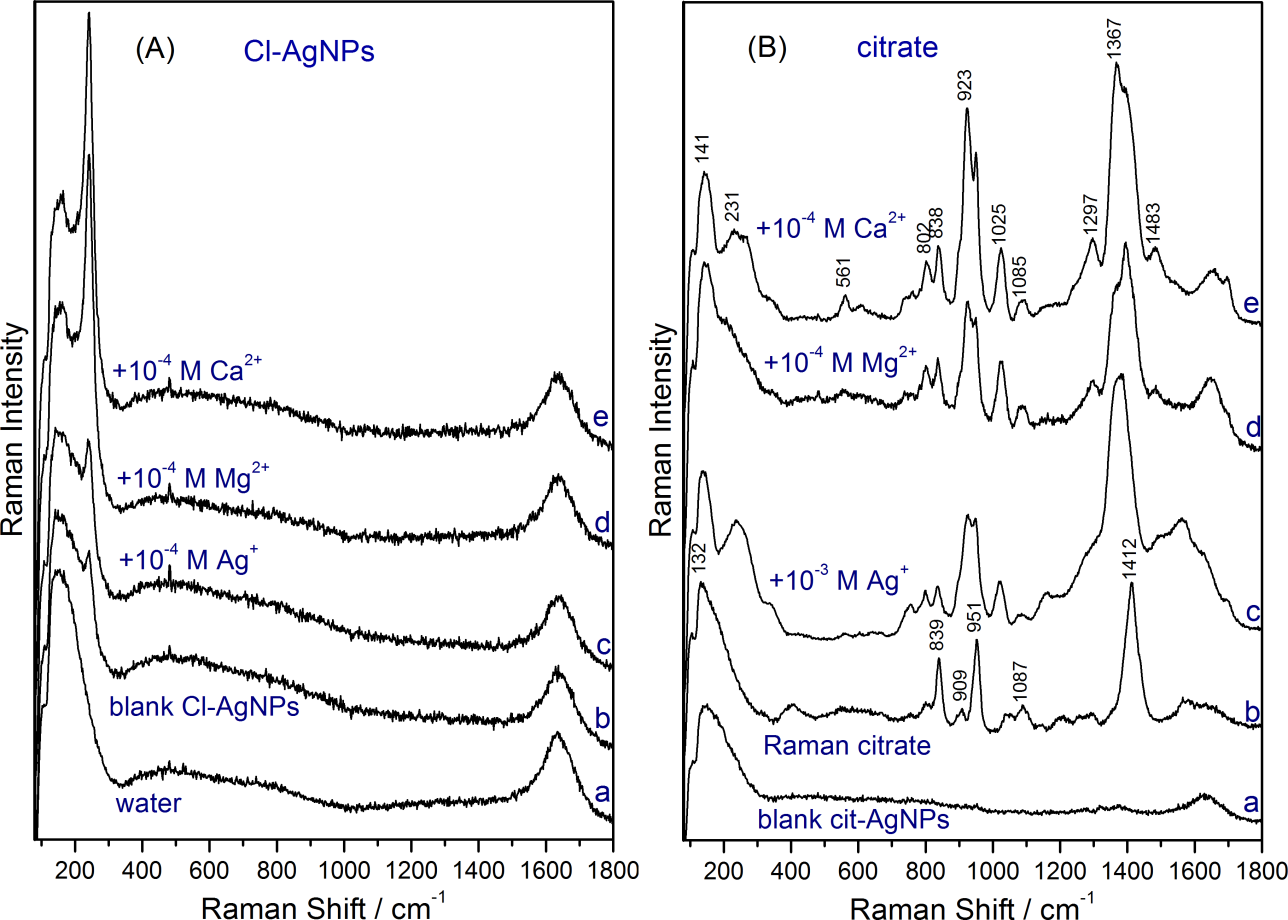
(A) Raman spectrum of water (a), SERS spectrum of Cl-AgNPs (b), SERS spectrum of Cl-AgNPs after addition of 10^−4^ M AgNO_3_ (c), 10^−4^ M MgSO_4_ (d), and 10^−4^ M Ca(NO_3_)_2_ (e) showing the intense Ag–Cl SERS band. (B) Raman blank spectrum of cit-AgNPs (a), Raman spectrum of 1 M citrate (b), SERS spectra of citrate obtained after addition of 10^−3^ M AgNO_3_ (c), 10^−4^ M MgSO_4_ (d), and 10^−4^ M Ca(NO_3_)_2_ (e) to the cit-AgNPs.

The Raman spectra of conventional silver colloids used as a SERS substrate contain no distinct peaks, since the concentration of the reagents (in the mM range) is too low for normal Raman spectroscopy. The surfactant (capping agent) plays a key role in the stabilization of the AgNPs by prohibiting aggregation via electrostatic repulsion between AgNPs. However, the surfactant surrounding the AgNPs does not exhibit a SERS spectrum if an electronic coupling with the silver surface is absent. Figure 5 shows the spectra of the Cl-AgNP and cit-AgNP colloids. For comparison, the Raman spectrum of water is given.

The blank SERS spectrum of Cl-AgNPs (Figure 5A) shows a very low intensity band at 242 cm^−1^ assigned to Ag–Cl vibration, indicating that a small proportion of Cl^−^ ions are chemisorbed onto the silver surface. These chemisorbed Cl^−^ ions form the SERS-active sites on the as-synthesized colloidal AgNPs which facilitate the recording of intense SERS spectra of cationic dye analytes (Figure 4). In contrast, the blank spectrum of cit-AgNPs (Figure 5B) shows a similar spectral shape to that of water, i.e., there are no SERS bands to indicate an activation of the cit-AgNPs. Thus, as shown in Figure 4, the as-synthesized cit-AgNPs colloid is SERS inactive for crystal violet 10^−8^ M and 9-aminoacridine 10^−7^ M.

The number of SERS-active sites on Cl-AgNPs can be increased considerably by promoting the chemisorption of more Cl^−^ ions onto the AgNP surface using cations such as Ag^+^, Mg^2+^ or Ca^2+^. Thus, by adding salts such as AgNO_3_, MgSO_4_ or Ca(NO_3_)_2_ at a concentration of 10^−4^ M to the Cl-AgNPs, an increase in the intensity of the Ag–Cl vibration band at 242 cm^−1^ is observed due to the additional chemisorption of Cl^−^ ions onto the silver surface mediated by the Ag^+^, Mg^2+^ or Ca^2+^ (Figure 5A). Ag^+^ 10^−4^ M ions induce only a slight increase of the number of SERS-active sites, as can be deduced from the slight intensity increase of the 242 cm^−1^ band. It should be mentioned that the Cl-AgNP colloid activated with Ag^+^, Mg^2+^ or Ca^2+^ 10^−4^ M is stable for weeks.

**Figure 6:**
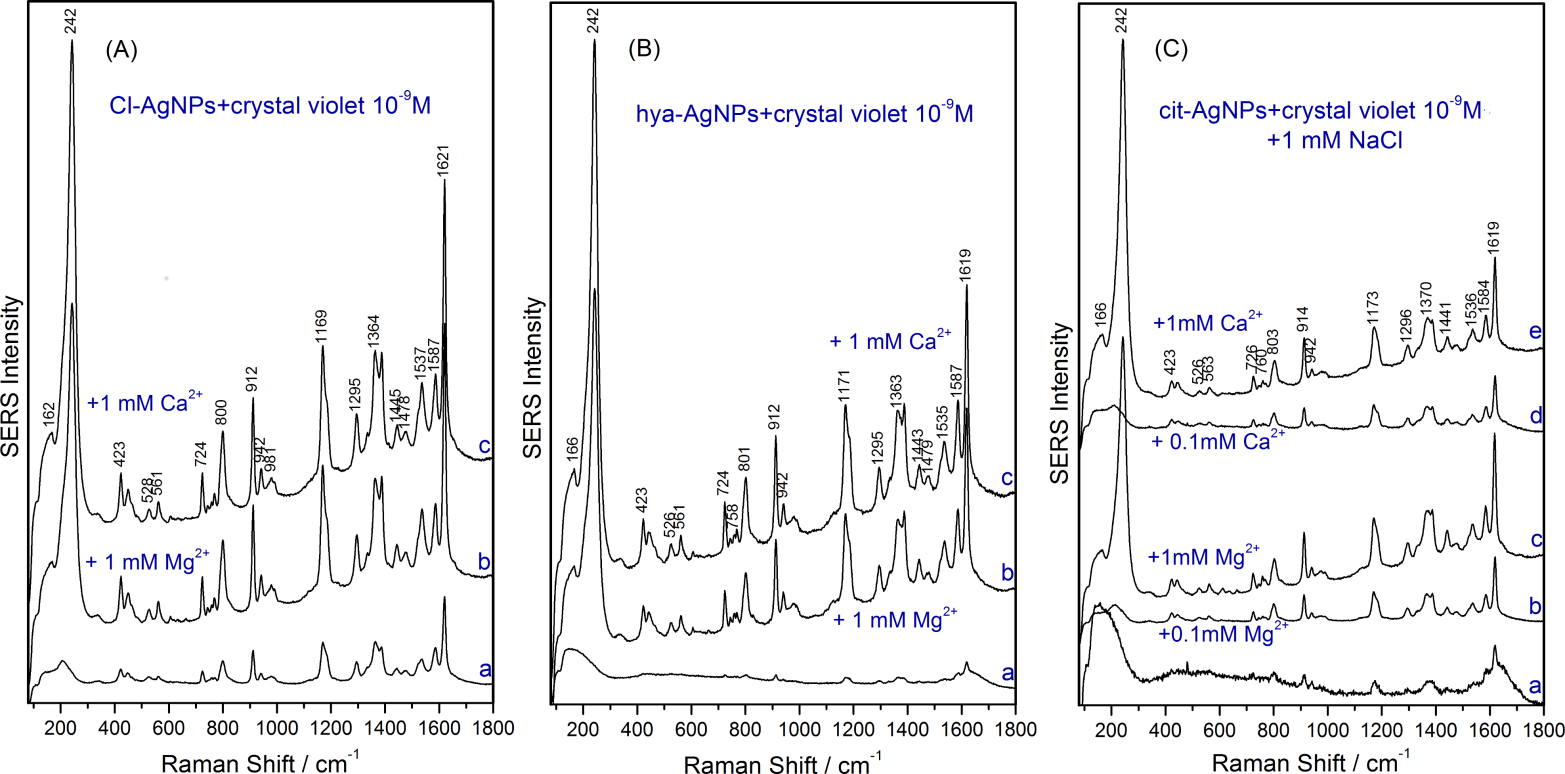
SERS spectra of 10^−9^ M crystal violet recorded using the following as substrates: (A) Cl-AgNPs (a), Cl-AgNPs and 1 mM MgSO_4_ (b), 1 mM Ca(NO_3_)_2_ (c); (B) hya-AgNPs (a), hya-AgNPs and 1 mM MgSO_4_ (b), 1 mM Ca(NO_3_)_2_ (c); (C) cit-AgNPs+1mM NaCl (a), cit-AgNPs+1mM NaCl and 10^−4^ M MgSO_4_ (b), cit-AgNPs+1mM NaCl and 1 mM MgSO_4_ (c), cit-AgNPs+1mM NaCl and 10^−4^ M Ca(NO_3_)_2_ (d), cit-AgNPs+1mM NaCl and 1 mM Ca(NO_3_)_2_ (e).

In line with the adatom model, the generation of additional SERS-active sites on the metal surface leads to an increase in the SERS intensity of analytes, as shown for 10^−9^ M crystal violet in Figure 6A. The addition of 1 mM MgSO_4_ or Ca(NO_3_)_2_ leads to a visibly increased SERS signal of crystal violet 10^−9^ M (roughly three times). Besides the intense SERS bands of the analyte, the intense Ag–Cl band at 242 cm^−1^ evidences the high number of SERS-active sites. However, in the case of Cl-AgNPs, the increase in intensity after generating additional SERS-active sites on the Cl-AgNP surface is not as dramatic, since most of the analyte molecules were already chemisorbed in the as-synthesized colloidal solution, due to their low concentration (10^−9^ M).

The increase in the SERS intensity of 10^−9^ M crystal violet is more impressive when using activated hya-AgNPs as a substrate, as shown in Figure 6B. The hya-AgNPs contain Cl^−^ ions in a concentration of 2.4 mM and the addition of cations such as Mg^2+^ or Ca^2+^ in concentrations of 1 mM mediates the chemisorption of more chloride ions onto the silver surface and the formation of more SERS-active sites. Thus, the very low intensity SERS spectrum of 10^−9^ M crystal violet, recorded with the as-synthesized hya-AgNPs, becomes a very intense SERS spectrum after the addition of MgSO_4_ or Ca(NO_3_)_2_ 1 mM (Figure 6B).

The lack of SERS-active sites in the as-synthesized cit-AgNPs allows the observation in an instructive manner of the SERS switch effect after SERS activation of colloid and the coupling of the analyte to the silver surface.

Since the as-synthesized cit-AgNPs did not allow the recording of SERS bands of 10^−9^ M crystal violet, we added 1 mM NaCl to activate the cit-AgNPs. However, the presence of 1 mM Cl^−^ led only to a weak activation of the cit-AgNPs, where the SERS intensity of 10^−9^ M crystal violet (Figure 6C) was comparable to that recorded with the as-synthesized hya-AgNPs (Figure 6B).

However, the further addition of MgSO_4_ or Ca(NO_3_)_2_ in concentrations ranging from 10^−4^ –10^−3^ M to a cit-AgNP solution which already contains 1 mM NaCl, led to the switching on of the SERS effect and the recording of very intense SERS spectra of 10^−9^ M crystal violet. Accordingly, the intense SERS spectra of crystal violet obtained after the generation of SERS-active sites on the surface of cit-AgNPs by adding 10^−3^ M Cl^−^ and 10^−4^ M Mg^2+^ or Ca^2+^ are shown in Figure 6C. Moreover, a further raise in intensity of the SERS spectra of crystal violet can be observed by increasing the concentration of Mg^2+^ or Ca^2+^ ions to 1 mM in the case of cit-AgNPs which were previously activated by adding 1 mM NaCl (Figure 6C).

In summary, high-intensity SERS spectra of cationic dyes can be recorded with all three silver colloids after activation. For this, our recommendation is that the silver colloid should contain Cl^−^ anions in a concentration range between 10^−3^–10^−2^ M and Mg^2+^ or Ca^2+^ in concentrations between 10^−4^–10^−3^ M.

In order to study the effect of the Cl^−^ surface density and dye concentration on Raman enhancement, we performed a series of SERS studies on R6G in the 10^−7^–10^−11^ M concentration range using Cl-AgNPs and cit-AgNPs, the details of which are included in Supporting Information File 1 (Figures S3 and S4).

In the case of R6G analyzed with as-synthesized Cl-AgNPs, the limit of detection was 10^−8^ M (Supporting Information File 1, Figure S3A, spectrum b). In other words, the number of Cl^−^ SERS-active sites on the silver surface is enough to observe the SERS spectrum of R6G down to a concentration of 10^−8^ M. However, the detection limit of R6G can be reduced to 10^−11^ M by adding Ca^2+^ ions, which promote the adsorption of additional Cl^−^ and thus increase the number of Cl^−^ SERS-active sites (Supporting Information File 1, Figure S3D, spectrum b and c). Although the absolute surface density of Cl^−^ is difficult to calculate, the relative surface density of Cl^−^ can be estimated based on the 240 cm^−1^ band of Ag–Cl. For example, when analyzing 10^−11^ M R6G with Cl-AgNPs, the results show that the intensity of the 240 cm^−1^ band increases from ≈50 counts when using 0.1 mM Ca^2+^ to about ≈250 counts for 1 mM Ca^2+^ (Supporting Information File 1, Figure S3D, spectrum b and c).

By increasing the Ca^2+^ concentration from 0.1 mM up to 1 mM in Cl-AgNPs, an increase of the number of SERS-active sites in the Cl-AgNPs was observed, as highlighted in Supporting Information File 1, Figure S3 by the increase of the intensity of the R6G SERS spectra (Figure S3A,B) or of the Ag–Cl band intensity (Figure S3C,D). Further increasing the Ca^2+^ concentration leads to the aggregation of the colloid and therefore the SERS spectrum of the analyte is not observable anymore.

In the case of 10^−7^, 10^−8^ and 10^−11^ M R6G analyzed with as-synthesized cit-AgNPs, only the fluorescence emission due to R6G was observed (Supporting Information File 1, Figure S4A,B). The SERS spectrum of R6G 10^−8^ M shows only fluorescence emission even after the addition of 10^−4^ M Ca^2+^ (Supporting Information File 1, Figure S4A, spectrum b). However, after the addition of 10^−3^ M Cl^−^ ions, the SERS spectrum of R6G is turned on (Supporting Information File 1, Figure S4A, spectrum c). An increase in the intensity of the SERS spectrum of R6G is observed after increasing the Cl^−^ concentration to 10 mM (Supporting Information File 1, Figure S4A, spectrum d), thus evidencing the increase in Cl^−^ SERS-active sites.

A detection limit of 10^−11^ M for R6G was obtained also with cit-AgNPs. However, an activation of the cit-AgNPs with 0.1 mM Ca(NO_3_)_2_ and 10 mM NaCl was necessary (Supporting Information File 1, Figure S4B, spectrum b).

Figure S4 in Supporting Information File 1 highlights an increase of SERS-active sites by increasing the Cl^−^ concentration up to 10 mM, clearly showing that the SERS intensity is proportional to the number of generated Cl^−^ adatoms up to a concentration of 10^−2^ M. Further increasing the Cl^−^ concentration leads to the aggregation of the colloid and therefore the SERS spectrum of the analyte is not observable anymore.

Therefore, these results suggest that the number of active SERS sites is the key in determining the enhancement factor and that nanoparticle geometry and capping agent due to different synthesis methods play secondary roles.

Similar to Cl^−^, the addition of Ag^+^, Mg^2+^ or Ca^2+^ to cit-AgNPs mediates the electronic coupling between citrate and the metallic silver surface (Figure 5B). Indeed, right after adding 1 mM AgNO_3_ or 10^−4^ M MgSO_4_ or Ca(NO_3_)_2_, the SERS effect is turned on and intense SERS spectra of citrate can be observed (Figure 5B) [30]. The main SERS bands of citrate in Figure 5B appear shifted towards lower wavenumbers compared to the Raman bands, indicating a chemical interaction between the citrate molecules and the silver surface.

Thus, the SERS bands of citrate previously reported by us [31–32] during the laser induced synthesis of SERS-active silver spots using silver nitrate-citrate mixtures can easily be explained by the presence of excess of Ag^+^ in the solution, which induces the chemisorption of citrate onto the metal silver surface.

Nevertheless, we could not obtain the SERS spectrum of citrate when using silver colloids which contain chloride (hya-AgNPs, Cl-AgNPs) or when using cit-AgNPs which were activated with Cl^−^ in concentrations higher than 1 mM. This is because the citrate molecules cannot displace chloride ions from the AgNP surface due to the higher affinity for the surface of the chloride ions compared to citrate.

The affinity between anions and the activated AgNP surface [18] also explains why we did not observe any SERS signal from NO_3_^−^ or SO_4_^2−^, which should exhibit characteristic bands at 1046 cm^−1^ and 980 cm^−1^, respectively [30,33] and are known to have a weak affinity for the silver surface. Therefore, NO_3_^−^ or SO_4_^2−^ cannot displace the more tightly adsorbed chloride or citrate molecules from the surface of the nanoparticles (Figure 5) [33].

Due to the specificity of the Ca^2+^ or Cl^−^ SERS-active sites, the SERS spectrum of the anionic or cationic analyte in the same solution can be turned on sequentially. Thus, by activating the cit-AgNPs with Ca^2+^ or Cl^−^, the SERS spectrum of citrate or the spectrum of cationic dye was recorded sequentially from their mixture. Figures S5 and S6 in Supporting Information File 1 show the specificity of SERS detection of citrate and R6G or crystal violet. A schematic representation of the specific chemisorption and SERS detection of citrate and crystal violet is shown in Supporting Information File 1, Figure S7.

Chloride-containing and chloride-free colloids can therefore lead to different SERS spectra. This feature was observed also for zwitterionic L-cysteine [34–36]. In view of the results presented here, L-cysteine is chemisorbed via its COO^−^ group to the silver surface at Ag^+^ SERS-active sites when using cit-AgNPs as a SERS substrate. However, in Cl^−^ activated colloids, L-cysteine is chemisorbed by its NH_3_^+^ group at Cl^−^ SERS-active sites.

## Conclusion

We described a simple protocol for synthesizing Cl-AgNPs with a mean diameter of 36 nm by photoconversion from AgCl precursor microparticles, in the absence of any organic reducing or capping agent. The as-synthesized Cl-AgNPs are already highly SERS-active due to chemisorbed Cl^−^ ions, a feature which renders the Cl-AgNPs more advantageous compared to the as-synthesized hya-AgNPs or cit-AgNPs for acquiring SERS spectra of cationic molecules.

Comparative SERS experiments employing Cl-AgNPs, hya-AgNPs and cit-AgNPs colloids, which contain different amounts of Cl^−^ ions, highlight fundamental aspects of SERS: the SERS effect is turned on only after the electronic coupling of the analyte to the silver surface at SERS-active sites.

The chemisorption of anionic species, like Cl^−^ or citrate, is mediated by cations such as Ag^+^, Mg^2+^, or Ca^2+^. The electronic coupling of cationic analytes to the silver surface occurs at SERS-active sites on the AgNP surface formed by chemisorbed Cl^−^ ions. Our preliminary studies indicate that chemisorbed I^−^ and Br^−^ anions also form SERS-active sites for cationic analytes.

The experiments presented in this study also suggest that the SERS-activation role of ions such as Cl^−^, Ag^+^, Mg^2+^ or Ca^2+^ is more important than the Raman enhancement due to nanoparticle aggregation. Thus, the generation of SERS-active sites is decisive for obtaining high intensity SERS spectra, this feature being more important than the size, geometry or the capping agent of the SERS substrate.

In summary, to fully exploit the enhancing property of colloidal AgNPs for SERS of cationic molecules, the silver colloid should contain Cl^−^ anions in a concentration range between 10^−3^–10^−2^ M and Mg^2+^ or Ca^2+^ cations in concentrations between 10^−4^–10^−3^ M. For the SERS detection of anionic analytes, chloride-free colloids should be considered.

## Experimental

Analytical grade silver nitrate (puriss. p.a., Sigma-Aldrich), sodium chloride (Reag. Ph. Eur., Merck), sodium hydroxide (50% w/w solution, Fluka Analytical), hydrogen peroxide (10% w/w solution, AppliChem), sodium citrate dihydrate (99%, Sigma-Aldrich), hydroxylamine hydrochloride (99%, Sigma-Aldrich), magnesium sulfate (99%, Sigma-Aldrich), calcium nitrate tetrahydrate (99%, Sigma-Aldrich), potassium iodide (99%, Sigma-Aldrich), crystal violet chloride (Reag. Ph. Eur., Merck), 9-aminoacridine hydrochloride monohydrate (for synthesis, Merck) and rhodamine 6G (99%, Sigma-Aldrich) were dissolved in ultrapure water (Direct-Q 3 UV, Millipore, resistivity higher than 18 MΩ).

### Synthesis of silver colloids

The synthesis of all silver colloids used in this study is described below. After synthesis, all colloids were stored in ambient conditions.

#### Silver nanoparticles obtained by citrate reduction (cit-AgNPs)

To obtain the cit-AgNPs, 17 mg of silver nitrate was solved in 98 mL ultrapure water and heated to boiling. Then, 0.020 g of citrate solved in 2 mL ultrapure water was added dropwise. The mixture was further boiled under vigorous stirring for 50 min.

#### Silver nanoparticles obtained by hydroxylamine hydrochloride reduction (hya-AgNPs)

To prepare the hya-AgNPs, 17 mg of silver nitrate were solved in 90 mL ultrapure water. In a separate container, 17 mg of hydroxylamine hydrochloride and 1.2 mL of sodium hydroxide solution (1%) were mixed in 10 mL ultrapure water. The silver colloid was obtained at room temperature by rapidly adding the hydroxylamine hydrochloride/sodium hydroxide mixture to the silver nitrate solution under vigorous stirring. The resulting colloid was further stirred for 5 min [3].

#### Chloride-capped silver nanoparticles obtained by photoreduction (Cl-AgNPs)

For the preparation of the new Cl-AgNPs, 40 mL of water was placed in a plastic beaker and the following reagents were added in the mentioned order: 400 μL of a 1 M sodium chloride solution, 180 μL of a 1% sodium hydroxide solution, 200 μL of a 10% hydrogen peroxide solution, 400 μL of a 0.1 M silver nitrate solution. The pH of the reaction mixture reduced from 10 to 7.5 during the synthesis.

The optimal amount of sodium chloride was determined from repeated synthesis experiments. Stable silver colloidal solutions were obtained by adding 1 M NaCl to the synthesis mixture at a volume between 300 and 500 μL.

After the addition of the reagents, the beaker was exposed to the light provided by a conventional desk lamp equipped with a commercial LED bulb (23 W, 6500 K, cool white, 2452 lm), under constant magnetic stirring (400 rotations/minute). Figure 1 shows a picture of the experimental setup and a picture of the resulting colloid.

For monitoring the photosynthesis of Cl-AgNPs, aliquots of 100 μL were taken from the reaction mixture at sequential time points during the synthesis reaction, diluted 10-fold, and analyzed by UV–vis absorption, as shown in Figure 2.

Prior to the electron microscopy analysis, a drop of colloidal solution was deposited and dried on a copper grid coated by a thin carbon film. The measurements were carried out using a Hitachi HD-2700 scanning transmission electron microscope (STEM), equipped with a cold field emission gun, working at an acceleration voltage of 200 kV.

The absorption spectra were recorded with a Jasco V-630 UV–vis spectrometer at 1 nm spectral resolution.

Raman and SERS spectra were obtained with a Renishaw InVia Raman microscope, equipped with a Nd:YAG frequency-doubled laser emitting at 532 nm and a laser power of ≈60 mW on the sample. For each measurement, 10 μL of the sample was placed on a microscope slide that was covered with aluminum foil. The laser light was focused on the drop using a 5× objective (NA 0.12). The spectra were recorded by averaging four acquisitions of 4 sec each.

## Supporting Information

File 1 Additional experimental data.
