# Supplementary material for: The role of adatoms in chloride-activated colloidal silver nanoparticles for surface-enhanced Raman scattering enhancement
Source: Beilstein J Nanotechnol. 2018 Aug 22;9:2236–47. doi: 10.3762/bjnano.9.208 (PMC6122276; doi:10.3762/bjnano.9.208)
Supplement: File 1 — Additional experimental data. [file Beilstein_J_Nanotechnol-09-2236-s001.pdf]

Supporting Information

for

The role of adatoms in chloride-activated colloidal silver nanoparticles for surface-enhanced Raman scattering enhancement

Nicolae Leopold*[1], Andrei Stefancu*[1], Krisztian Herman[1], István Sz. Tódor*[1], Stefania D. Iancu[1], Vlad Moisoiu[1], and Loredana F. Leopold*[2]

Address: [1]Faculty of Physics, Babeş-Bolyai University, Kogalniceanu 1, 400084 Cluj-Napoca, Romania and [2]Faculty of Food Science and Technology, University of Agricultural Sciences and Veterinary Medicine, Manastur 3-5, 400372 Cluj-Napoca, Romania

Email: Nicolae Leopold - nicolae.leopold@phys.ubbcluj.ro; Loredana Florina Leopold - loredana.leopold@usamvcluj.ro

* Corresponding author

Additional experimental data

Content

- UV-Vis absorption of the individual reagent solutions used for the preparation of Cl-AgNPs
- UV-Vis spectra obtained during the Cl-AgNPs photosynthesis, without addition of hydrogen peroxide and evidence of SERS activity of Cl-AgNPs
- Concentration dependent SERS spectra of R6G
- Specific SERS detection

UV-Vis absorption of the individual reagent solutions used for the preparation of Cl-AgNPs

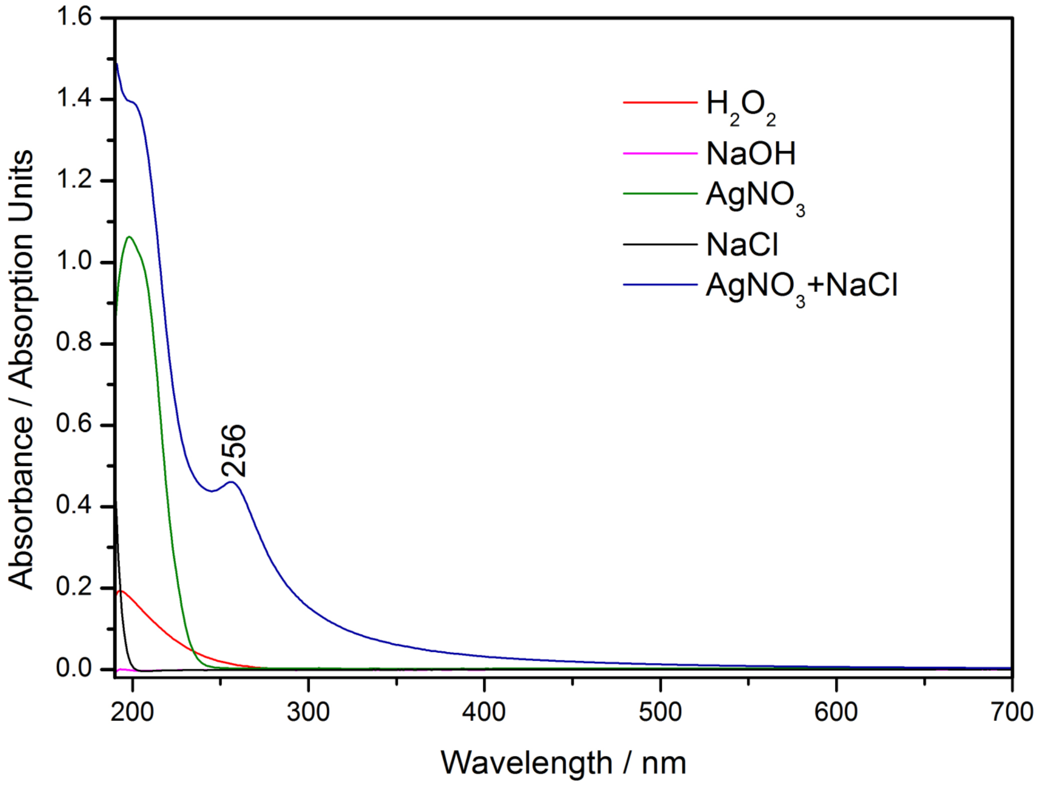

Figure S1: UV-Vis absorption of the individual reagent solutions, as indicated in the figure, used for the preparation of Cl-AgNPs. Individual reagent amounts and concentrations as used for Cl-AgNPs synthesis were added to 40 ml water and diluted 10-fold for the recording of the UV-Vis absorption spectra. A new absorption band arises at 256 nm after the mixing of $AgNO_3$ with NaCl solutions, assigned to the formed AgCl complexes.

UV-Vis spectra obtained during the Cl-AgNPs photosynthesis, without addition of hydrogen peroxide and evidence of SERS activity of Cl-AgNPs

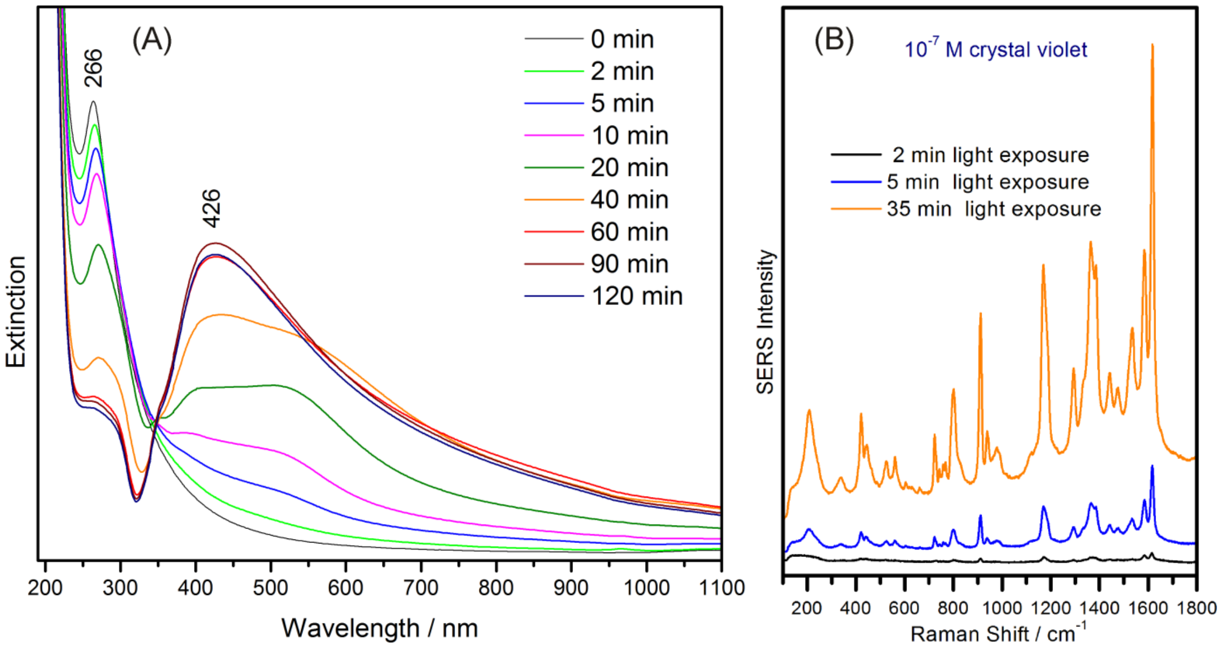

Figure S2: (A) UV-Vis spectra obtained during the Cl-AgNPs photosynthesis, without addition of hydrogen peroxide to the synthesis reaction, the light exposure times being indicated in the figure; **(B)** SERS activity of the Cl-AgNPs obtained without addition of hydrogen peroxide to the synthesis reaction. The recorded SERS spectra of 10^{-7} M crystal violet using Cl-AgNPs obtained after 2, 5 and 35 minutes of light exposure.

Concentration dependent SERS spectra of R6G

In the case of R6G analysed with as-synthesized Cl-AgNPs, intense SERS spectra of R6G could be obtained at a concentration of 10^{-7} M (Figure S3 A, spectrum a), the limit of detection being 10^{-8} M (Figure S3 A, spectrum b).

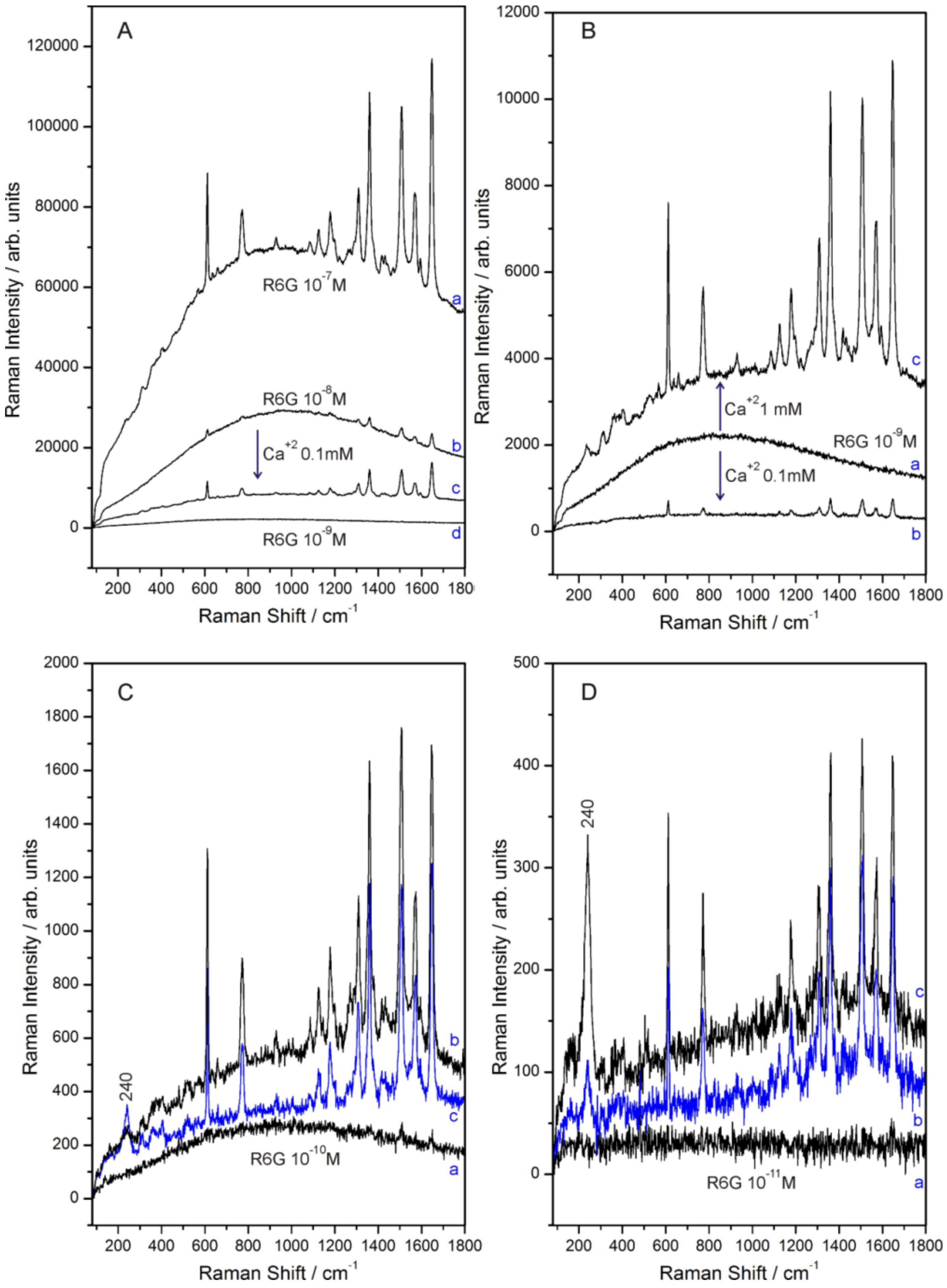

Figure S3. Concentration-dependent SERS spectra of R6G using Cl-AgNPs as substrate. **A:** R6G 10^{-7} M (a), R6G 10^{-8} M (b), R6G 10^{-8} M and $Ca(NO_3)_2$ 0.1 mM (c), R6G 10^{-9} M (d); **B:** R6G 10^{-9} M (a), R6G 10^{-9} M and $Ca(NO_3)_2$ 0.1 mM (b), R6G 10^{-9} M and $Ca(NO_3)_2$ 1 mM (c); **C:** R6G 10^{-10} M (a), R6G 10^{-10} M and $Ca(NO_3)_2$ 0.1 mM (b), R6G 10^{-10} M and $Ca(NO_3)_2$ 1 mM (c); **D:** R6G 10^{-11} M (a), R6G 10^{-11} M and $Ca(NO_3)_2$ 0.1 mM (b), R6G 10^{-11} M and $Ca(NO_3)_2$ 1 mM (c). Experimental conditions:

laser power 1.2 mW on the sample, exposure time 1s, 4 acquisitions. The figures show the raw spectra, as recorded.

The SERS spectrum of R6G 10^{-8} M acquired with as-synthesized Cl-AgNPs (Figure S3 A, spectrum b) is dominated by a fluorescence background, due to the 'free', unadsorbed molecules. However, the addition of $Ca(NO_3)_2$ 10^{-4} M promotes the adsorption of additional Cl^- ions, which form SERS active sites for R6G molecules. Consequently, the SERS spectrum of R6G 10^{-8} M becomes more intense, while the fluorescence background becomes much lower (Figure S3 A, spectrum c).

When analysing R6G in a concentration of 10^{-9} (Figure S3 A, spectrum d and Figure S3 B, spectrum a) and 10^{-10} M (Figure S3 C, spectrum a) with the as-synthesized Cl-AgNPs, no SERS bands of R6G are observed, the spectra being dominated by fluorescence emission. However, the addition of Ca^{2+} 10^{-4} M leads to the generation of additional SERS active sites, which promote the chemisorption of R6G and increases the intensity of the R6G SERS signal above the detection limit, while also decreasing the fluorescence background (Figure S3 B, spectrum b).

By further increasing Ca^{2+} concentration from 10^{-4} M to 10^{-3} M, an additional enhancement for R6G 10^{-9} M is observed (Figure S3 B, spectrum c). However, in the case of R6G 10^{-10} M, the SERS spectra obtained with Ca^{2+} 0.1 mM and 1 mM show similar intensities (Figure S3 C, spectrum b and c), indicating that the number of SERS active sites generated with 0.1 mM Ca^{2+} is enough for the chemisorption of all R6G molecules. Of course, the SERS spectrum of R6G 10^{-10} M without Ca^{2+} is blank (Figure S3 C, spectrum a).

As expected, the SERS spectrum of R6G 10^{-11} M acquired with the as-synthesized Cl-AgNPs is blank, despite the fluorescence emission being very weak (Figure S3 D, spectrum a). The addition of Ca^{2+} 0.1 mM turns on the SERS spectrum of R6G 10^{-11} M (Figure S3 D, spectrum b). As expected, a further increase in the Ca^{2+} concentration to 1 mM does not enhance the R6G SERS spectrum significantly, even if an increase in the number of Cl^- SERS active sites is evidenced by the Ag-Cl band at 240 cm^{-1} (~50 counts for 0.1 mM Ca^{2+} and ~250 counts for 1 mM Ca^{2+}, Figure S3 D, spectrum c). Therefore, these results suggest that for R6G in a concentration below 10^{-10} M, Ca^{2+} 0.1 mM is enough for the chemisorption all R6G

molecules, leading to a sensitivity of SERS spectroscopy which is comparable to that of fluorescence.

The SERS spectrum of R6G shows only fluorescence emission when using as-synthesized cit-AgNPs as substrate (Figure S4).

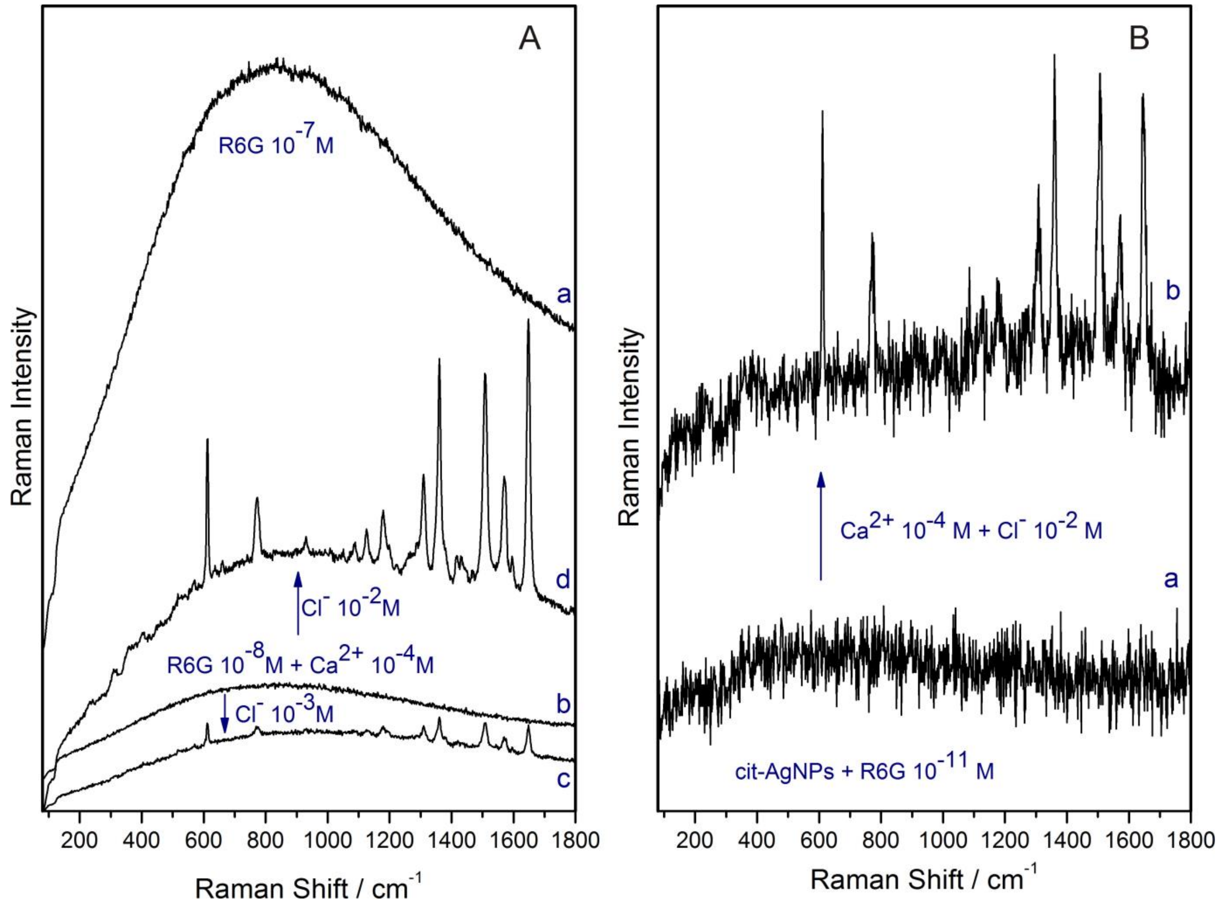

Figure S4. Concentration-dependent SERS spectra of R6G using cit-AgNPs as substrate. **A:** R6G 10^{-7} M (a), R6G 10^{-8} M and $Ca(NO_3)_2$ 0.1 mM (b), R6G 10^{-8} M, $Ca(NO_3)_2$ 0.1 mM and NaCl 1 mM (c), R6G 10^{-8} M, $Ca(NO_3)_2$ 0.1 mM and NaCl 10 mM (d); **B:** SERS spectra of R6G 10^{-11} M using cit-AgNPs as substrate and additional activation of the colloid with Ca^{2+} and Cl^-. Blank spectrum obtained for R6G 10^{-11} M in cit-AgNPs (a), SERS spectrum of R6G 10^{-11} M after activation of the colloid with $Ca(NO_3)_2$ 0.1 mM and NaCl 10 mM (b).

However the SERS spectrum of R6G is turned on by additional activation of the colloid with Ca^{2+} and Cl^-.

Specific SERS detection

The spectral table depicted in Figure S5 contains spectra recorded from the same R6G 10^{-11} M in cit-AgNPs solution, modified by sequentially adding Ca^{2+} and Cl^{-}, as shown in the Scheme S1.

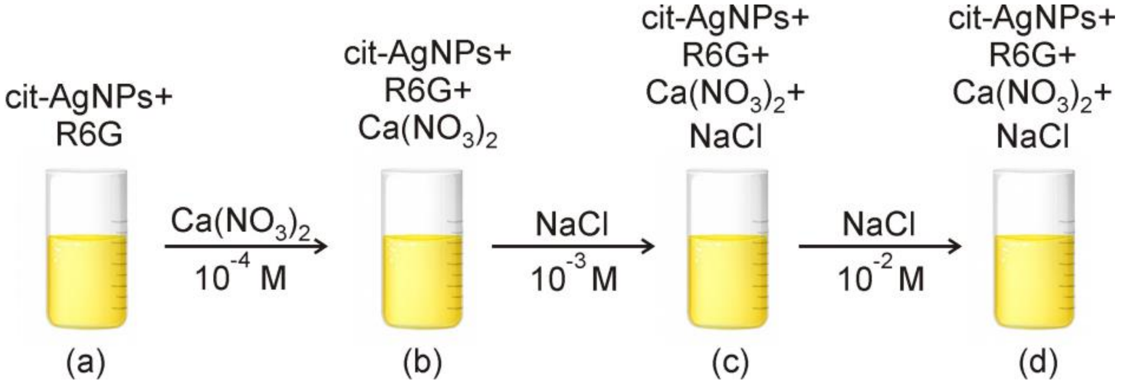

Scheme S1. Schematic illustration for the sequential addition of $Ca(NO_3)_2$ 0.1 mM and NaCl 1 mM and 10 mM to R6G 10^{-11} M in cit-AgNPs, as indicated.

Figure S5 shows the SERS spectra recorded in each of the situations depicted in Scheme S1.

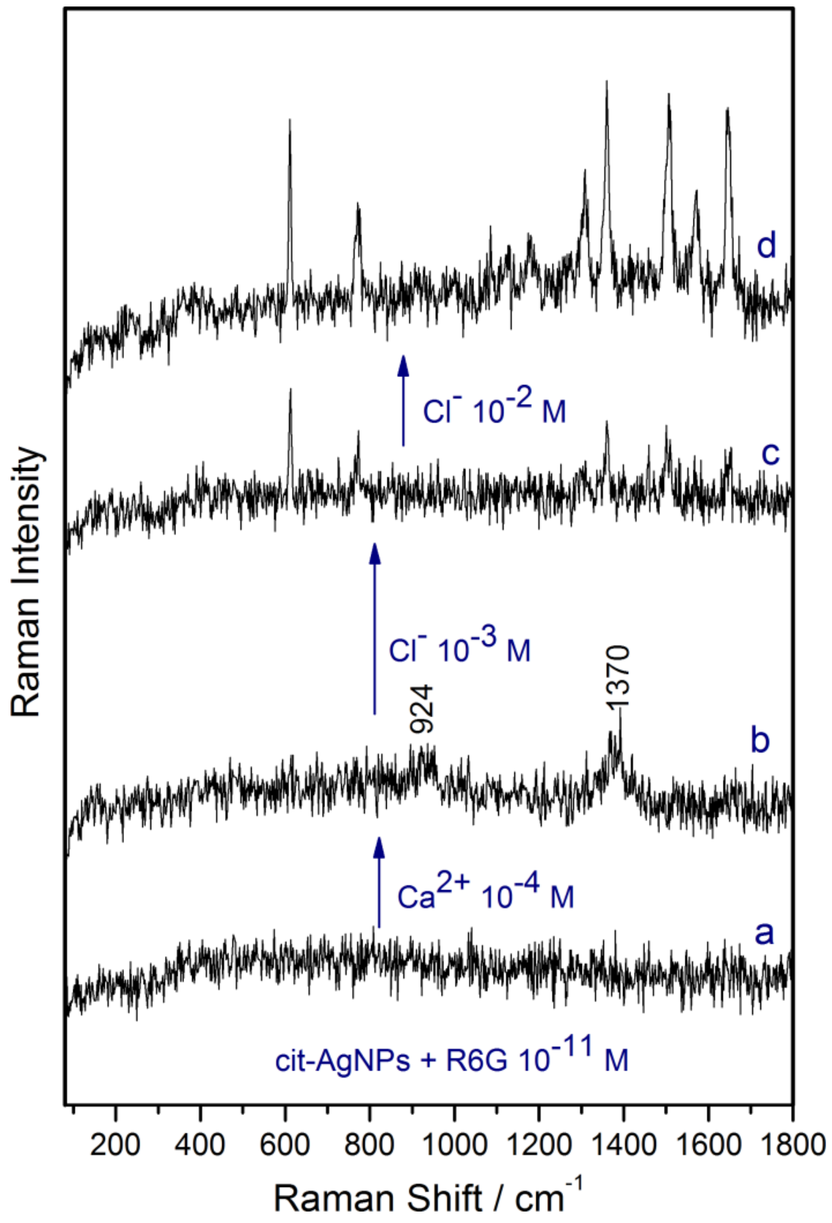

Figure S5. Specific SERS detection of citrate anion and R6G cationic dye by SERS activation of the cit-AgNPs with Ca^{2+} and Cl^-, respectively. Blank spectrum obtained for R6G 10^{-11} M in cit-AgNPs (a), SERS bands of citrate after activation of the colloid with Ca^{2+} 0.1 mM (b), SERS bands of R6G after addition of Cl^- 1 mM (c), SERS spectra of R6G after addition of Cl^- 10 mM (d). Experimental conditions: laser power 1.2 mW on the sample, exposure time 1s, 4 acquisitions.

The SERS spectrum of R6G 10^{-11} M with cit-AgNPs is blank (Figure S5, spectrum a). When to the same solution, $Ca(NO_3)_2$ 10^{-4} M is added, the recorded spectrum shows the two main citrate SERS bands at 924 and 1370 cm^{-1} (Figure S5, spectrum b),

indicating that the Ca^{2+} SERS active sites promote the chemisorption of citrate anions.

Next, when NaCl 10^{-3} M is added to the same solution, the SERS bands of citrate disappear, while the SERS bands of R6G begin to appear (Figure S5, spectrum c). Thus, the Cl^- ions, due to their higher affinity for the silver surface, replace the citrate anions from the Ca^{2+} SERS active sites, forming now Cl^- SERS active sites for R6G. Finally, the increase in the Cl^- concentration from 10^{-3} to 10^{-2} M leads to a further increase in the SERS intensity of R6G (Figure S5, spectrum d), clearly showing that the SERS intensity is proportional to the number of generated Cl^- adatoms for concentrations of Cl^- up to 10^{-2} M. By further increasing the Cl^- concentration, the colloid becomes aggregated and the SERS spectrum of the analyte disappears.

A similar behaviour is observed when using crystal violet 10^{-8} M and cit-AgNPs as substrate, proving once again the role of Cl^- ions in generating SERS active sites for crystal violet and in turning on of the SERS spectrum of crystal violet. Moreover, an increase in the SERS intensity of crystal violet is observed after the addition of Cl^- up to 10^{-2} M.

However, without the addition of Cl^- ions, we could not record any SERS spectra from R6G 10^{-11} M or crystal violet 10^{-8} M.

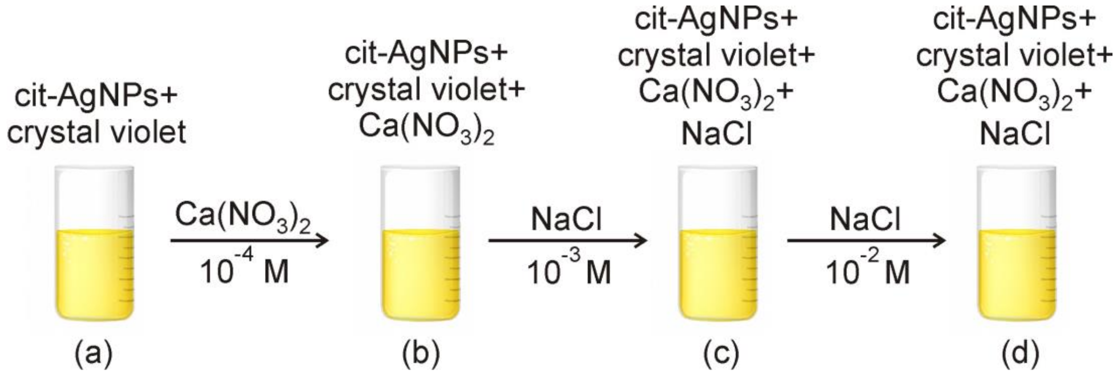

Scheme S2. Schematic illustration for the sequential addition of $Ca(NO_3)_2$ 0.1 mM and NaCl 1 mM and 10 mM to crystal violet 10^{-8} M in cit-AgNPs, as indicated.

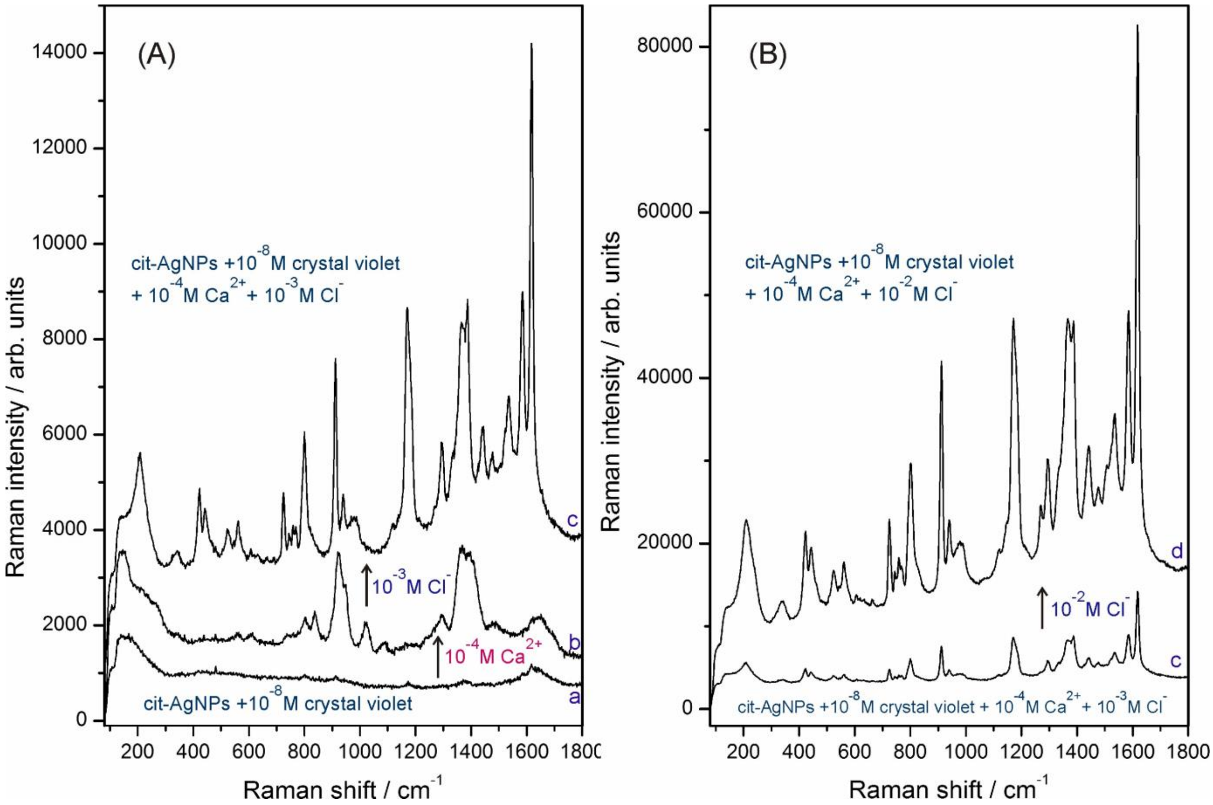

Figure S6. Specific SERS detection of citrate anion and crystal violet cationic dye by SERS activation of the cit-AgNPs with Ca^{2+} and Cl^-, respectively. **A:** Blank spectrum obtained for crystal violet 10^{-8} M in cit-AgNPs (a), SERS spectrum of citrate after activation of the colloid with Ca^{2+} 0.1 mM (b), SERS spectrum of crystal violet after addition of Cl^- 1 mM (c); **B:** SERS spectrum of crystal violet after addition of Cl^- 1 mM (c), SERS spectrum of crystal violet after addition of Cl^- 10 mM (d).

The SERS spectrum of crystal violet 10^{-8} M with cit-AgNPs is blank (Figure S6 A, spectrum a). The addition of Ca^{2+} 10^{-4} M turns on the SERS spectrum citrate (Figure S6 A, spectrum b). Next, the addition of Cl^- 10^{-3} M determines the replacement of the citrate anions by Cl^- anions at the Ca^{2+} SERS active sites, forming thus Cl^- SERS active sites for the cationic dye. Consequently, intense SERS spectra of crystal violet are recorded (Figure S6 A and B, spectrum c). Moreover, increasing Cl^- concentration up to 10^{-2} M leads to a further increase in the SERS intensity of crystal violet (Figure S6 B, spectrum d).

Figure S7 illustrates schematically the obtaining of the SERS spectra depicted in Figure S6, particularly the generation of Ca^{2+} SERS active sites, the chemisorption of

citrate and the generation of Cl^- SERS active sites, which then promote the chemisorption of crystal violet.

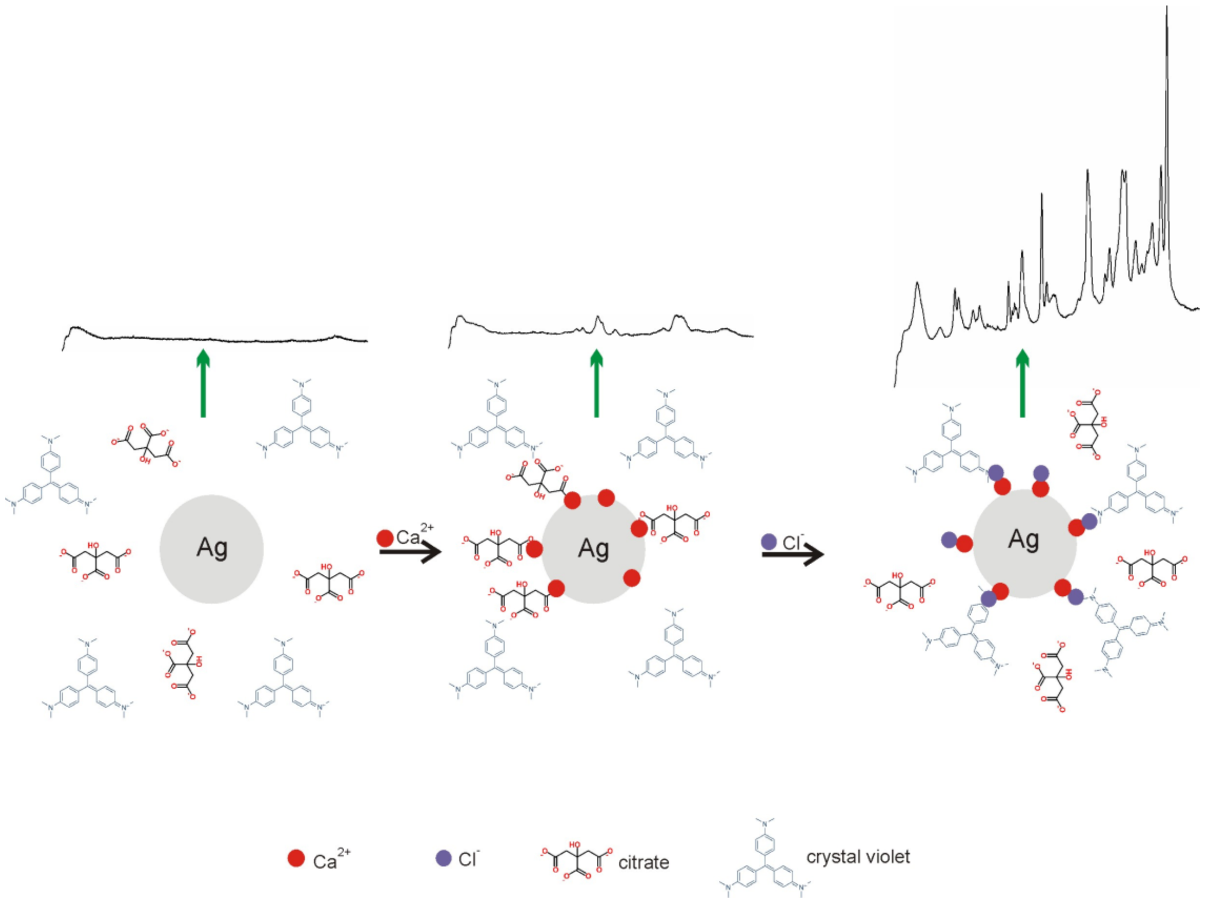

Figure S7. Schematic illustration for the obtaining of the SERS spectra depicted in Figure S6, after the SERS activation of the cit-AgNPs with Ca^{2+} and Cl^- ions.

In the absence of Ca^{2+} and Cl^- ions, there is no electronic contact between the silver surface and citrate or crystal violet molecules, the resulting Raman spectrum being similar to that of water.

The addition of Ca^{2+} promotes the chemisorption of citrate anions, thus the SERS spectrum of citrate was obtained.

Further addition of Cl^- ions determines the replacement of citrate from the Ca^{2+} SERS active sites by Cl^-, due to their higher affinity for the silver surface. The Cl^- ions form SERS active sites for crystal violet cationic dye, thus the SERS spectrum crystal violet is obtained.
